# Development of a novel hyaluronic acid membrane for the treatment of ocular surface diseases

**DOI:** 10.1038/s41598-021-81983-1

**Published:** 2021-01-27

**Authors:** Dong Ju Kim, Mi-Young Jung, Ha-Jin Pak, Joo-Hee Park, Martha Kim, Roy S. Chuck, Choul Yong Park

**Affiliations:** 1 Department of Ophthalmology, Dongguk University, Ilsan Hospital, 814, Siksadong, Ilsan-dong-gu, Goyang, Kyunggido 410-773 South Korea; 2 Department of Biochemistry, Dongguk University, College of Medicine, Gyeongju, South Korea; 3 Department of Ophthalmology and Visual Sciences, Montefiore Medical Center, Albert Einstein College of Medicine, Bronx, NY USA

**Keywords:** Conjunctival diseases, Corneal diseases

## Abstract

Ocular surface diseases (OSD) can cause serious visual deterioration and discomfort. Commercial artificial tear solution containing hyaluronic acid (HA) show excellent biocompatibility and unique viscoelastic characteristics. Here, we developed a novel HA membrane (HAM) by chemical crosslinking using 1,4-butanediol diglycidyl ether for the effective treatment of OSDs. The main purpose of HAMs is to provide sustained release of HA to modulate the wound healing response in OSDs. The safety and efficacy of HAMs were investigated using primary cultured human corneal epithelial cells and various OSD rabbit models. In the dry state, the HAM is firm, transparent, and easy to manipulate. When hydrated, it swells rapidly with high water retention and over 90% transmission of visible light. Human corneal epithelial cells and rabbit eyes showed no toxic response to HAM. Addition of HAMs to the culture medium enhanced human corneal epithelial cell viability and expression of cell proliferation markers. Investigation of HAM wound healing efficacy using mechanical or chemical corneal trauma and conjunctival surgery in rabbits revealed that application of HAMs to the ocular surface enhanced healing of corneal epithelium and reduced corneal limbal vascularization, opacity and conjunctival fibrosis. The therapeutic potential of HAMs in various OSDs was successfully demonstrated.

## Introduction

The ocular surface is the first anatomical structure to initiate the process of light perception, and it maintains optical clarity through anatomic, physiologic, and immunologic mechanism^1^. Unstable ocular surface, regardless of cause, can therefore lead to various forms of ocular surface diseases (OSDs) and cause ocular discomfort, dryness, and visual disturbance. The management of OSDs requires the control of the inflammation and restoration of the normal defense system by rapid healing of the ocular surface. In clinical ophthalmology, artificial tears are the mainstay of current therapy for the management of OSDs, and hyaluronic acid (HA) is one of the most popular components of commercial artificial tear eye drops^2^.

HA is a naturally occurring, endogenous, anionic glycosaminoglycan (GAG) polymer with repeating disaccharide units of d-glucuronic acid and *N*-acetyl-glucosamine^2,3^. It has cell proliferation, anti-inflammation, and antioxidant properties, and it functions in wound repair^4–7^. HA regulates various intracellular signaling and cell behaviors by binding to specific cell-surface receptors, such as the cluster of differentiation 44 (CD44), receptor for hyaluronan-mediated motility (RHAMM), lymphatic vessel endothelial hyaluronan receptor 1 (LYVE-1), and the liver endothelial cell (LEC) receptor^8^. Previous researches revealed that HA has molecular weight dependent roles in various phase of wound healing^9–11^.

The existence of the CD44 receptor on the human cornea and conjunctiva was reported in both normal and pathologic states^12,13^. In the normal cornea, CD44 is expressed in epithelial cells, keratocytes, and vascular endothelial cells^14,15^. However, in a pathologic condition, CD44 expression was increased in the corneal epithelium during healing^16^. HA application to the ocular surface increased the remodeling of extracellular matrix and resulted in a more organized collagen deposition at the wound site^17,18^. HA also has unique viscoelastic properties and shows a high water-retention capacity that enables improvement of ocular surface hydration and lubrication, thereby reducing surface friction^3,19,20^. Therefore, HA-containing artificial tears can facilitate the normalization of the corneal and conjunctival epithelium in patients with OSDs^12,20^.

The most common HA formulation for ophthalmic use is eye drops. Although there is a report that about 50% of high molecular weight HA eye drops persisted on the ocular surface 67 min after instillation^21^, the residence time of most topically applied eye drops on the ocular surface is usually short and disappears almost completely in less than 10 min after instillation; therefore, frequent instillations are needed to achieve a therapeutic effect^22–25^. Frequent instillation of eye drops is problematic for severe OSDs, such as in patients with burn wounds that require high therapeutic concentrations, as these patients may not be able to use eye drops properly without assistance. Therefore, more optimal treatment modalities are needed that can overcome the drawbacks of eye drops by increasing their retention time while promoting wound healing.

One way to reduce the frequency of polymer eye drop instillation is to increase the polymer rigidity by crosslinking^26,27^ as this prolongs polymer permanence at the clinical site and renders the polymer resistant to enzymatic degradation^28,29^. Several studies have shown that a cross-linked HA gel or membrane can accelerated corneal wound healing in alkali burn animal models^23,30^. In the present study, we used chemical cross-linking with 1,4-butanediol diglycidyl ether (BDDE) to formulate a novel HA membrane (HAM) for ocular surface application. The HAM is easy to apply, and it fits to the corneal curvature to provide a sustained therapeutic effect. Here, the physical characteristics and the ocular safety and efficacy of HAM were investigated in primary culture of corneal cells and in animal models.

## Results

### HAM formation and physical property

**Figure 1:**
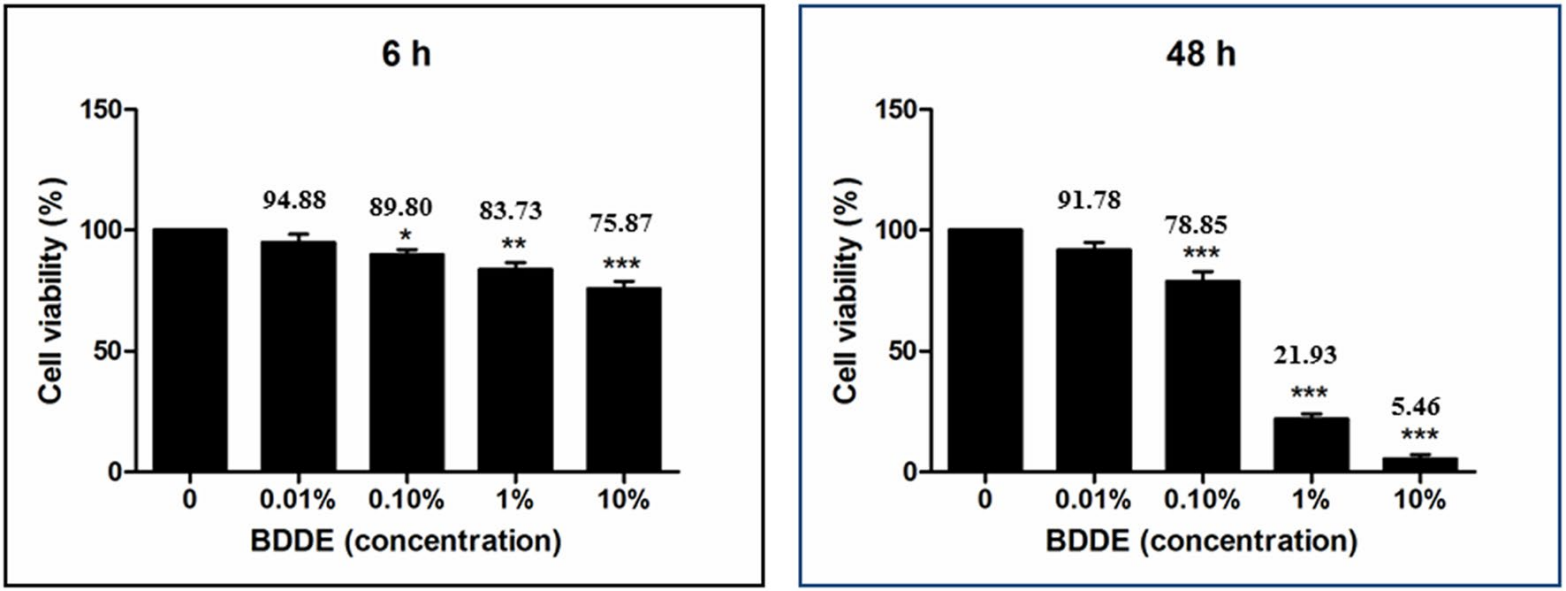
Primary human corneal epithelial cell viability following exposure to various concentrations of 1,4-butanediol diglycidyl ether (BDDE). Primary human corneal epithelial cells (HCECs) were exposed to different concentrations of BDDE for 6 and 48 h, and the cell viability was measured using CCK-8 analysis. A low concentration (0.01%) of BDDE had no significant toxic effect on the HCECs. Medium and high concentrations of BDDE induced significant HCEC toxicity, even after 6 h incubation, and a marked decrease (about 95%) in viability was observed following exposure to BDDE concentrations greater than 1% for 48 h.

The HAM was produced by cross-linking HA using BDDE. Dose-dependent BDDE toxicity tests using human corneal epithelial cells (HCECs) (Fig. 1) revealed that a low concentration (0.01%) of BDDE had no toxic effects on the HCECs at 6 and 48 h. A medium concentration (0.1%) of BDDE also showed no significant toxicity at 6 h; however, death of about 20% of the cells occurred after 48 h of incubation. A marked decrease in viability (over 70% cell death) was observed following exposure to high concentrations (1 and 10%) of BDDE for 48 h.

### Characterization of HAM

#### Water absorption capacity and optical transmittance

**Figure 2:**
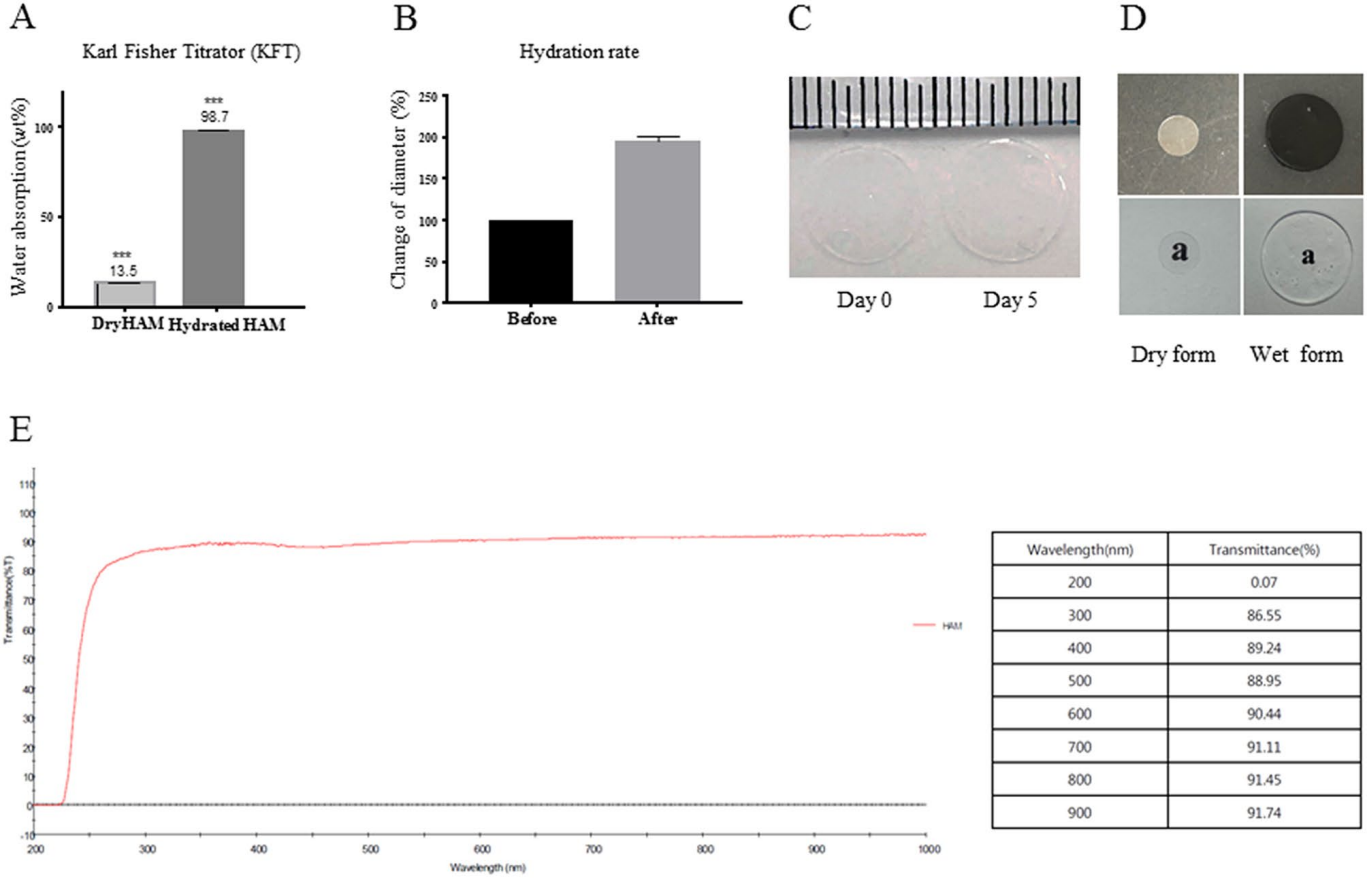
Physical properties of the HAM. (**A**) The water absorption capacity of the HAM was measured using a Karl Fisher Titrator (KFT). Dry HAM circles with a diameter 5 mm were hydrated in normal saline for 5 min. After hydration, the water content of the HAM increased from 13.5 to 98.7% (**B**,**C**) Swelling rate; Based on a 5 mm circle of the dry HAM, the diameter increased about twofold. Day 0 photo was taken after 5 min hydration and the increased diameter was similar until day 5. A letter underneath the HAM is clearly visible. (**D**,**E**) The optical transmittance of the HAM was over 90% for the most visible light wavelengths.

The water content of the dry HAM was 13.5% but increased to 98.7% after hydration (Fig. 2A). The swelling rate of the HAM was measured as a size change, based on a 5 mm circle of dry HAM. After reaching an equilibrium state, the size of the HAM increased about twofold compared to the dry HAM and remained unchanged until day 5 (Fig. 2B,C). The light transmittance of the hydrated and dry HAM was excellent and a letter underneath the membrane was fully legible (Fig. 2D). The optical transmittance of the hydrated HAM was evaluated at different wavelengths (200–900 nm) using a UV–Vis scanning spectrometer (Fig. 2E). The transmittance of visible light by the HAM was about 89–91% when hydrated.

#### Scanning electron microscope (SEM)

**Figure 3:**
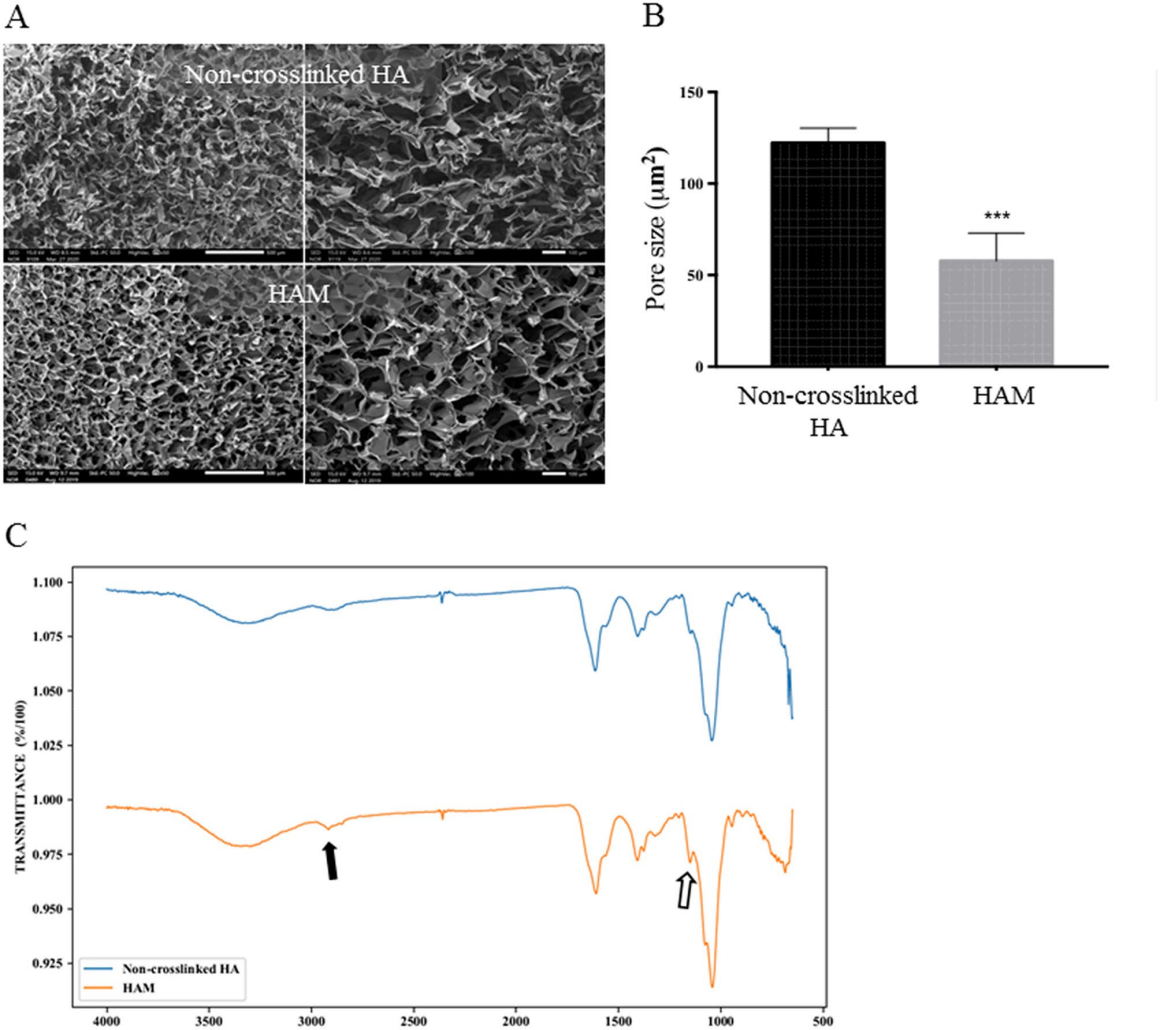
Morphological structure and chemical modification of the HAM. (**A**) Scanning electron microscopic images of non-crosslinked dried hyaluronic acid (HA) and crosslinked hyaluronic acid membrane (HAM). The HAM showed regular and dense porosity compared to non-crosslinked HA. (**B**) Scale bar indicates 100, 500 μm. The pore size of the HAM was significantly smaller than non-crosslinked HA. ***p < 0.001. (**C**) FT-IR (Fourier-transform infrared spectroscopy); Spectroscopic analysis of non-crosslinked HA and the HAM via FT-IR for conformation of cross-linkages. Two interesting new peaks were observed in the HAM spectrum: a new peak at 2900 cm^−1^ (black arrow) represented C–H stretching in the cross-linker and another new peak at 1300 cm^−1^ (empty arrow), which was assigned to an ether bond created after the combination of the hydroxyl group of HA and the epoxide group of BDDE.

SEM images showed information about the porosity and scaffold connectivity of the HAM. All samples were lyophilized before processing. The structures of non-crosslinked HA and HAM were compared by analyzing SEM images at identical magnification conditions (50×, 100×, SEI and 15 kv). The HAM showed a distinct difference in microstructure compared to non-crosslinked HA (Fig. 3A,B). Compared to non-crosslinked HA, the HAM showed a dense scaffold connectivity with higher porosity. Furthermore, the pore size of the HAM was smaller and more regularly distributed in comparison to non-crosslinked HA.

#### Fourier-transformed infrared (FT-IR) analysis

FT-IR is one of the most widely used techniques for analyzing the type of chemical composition formed during cross-linking^31,32^. The HAM showed the typical peak pattern for the cross-linking (Fig. 3C). The peak observed at around 2900 cm^−1^ in the HAM represented the C–H stretching after cross-linking^33,34^. Another additional small peak at about 1300 cm^−1^ in the HAM also confirmed the successful cross-linking process. This peak indicates the formation of ether bond as a result of HA chains and BDDE molecules^33,34^.

#### Detection of residual BDDE

**Figure 4:**
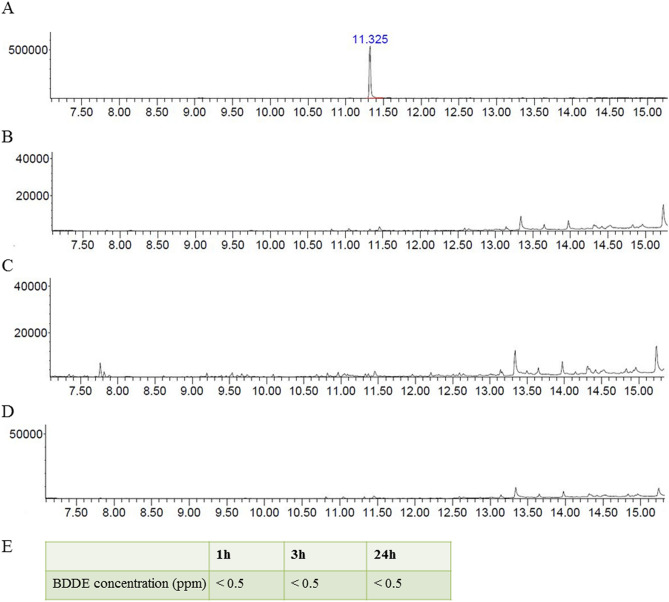
Gas chromatogrpahy-mass spectrometry (GC–MS) detection of BDDE using two different solvents. (**A**) A standard reference peak time of BDDE. To prepare the reference solution, BDDE was dissolved in saline solution at 2 ppm, which followed the safety recommendation of US Food and Drug Administration (FDA). A standard reference of peak was found at 11.325 min. (**B**–**D**) HAM was eluted in MEOH for 1 h (**B**), in saline for 3 h (**C**), in saline for 24 h (**D**). And the reference and HAM solutions were analyzed using GC–MS. HAM (**B**–**D**) showed no peak at 11.325 min. regardless of solvent and duration. (**E**) All measured BDDE were less than 0.5 ppm. These measurements satisfied the safety recommendation of US FDA for medical device.

The residual BDDE in the HAM was quantified using GC–MS to agree with FDA safety risk assessment. The level of residual BDDE should be below limit of 2 parts per million (ppm) for safe human application. A reference standard peak is 11.325 in Fig. 4A. Regardless of solvent and elution time, all results are under 0.5 ppm (Fig. 4B–E).

#### Analysis of molecular weight released from hydrolyzed HAM

**Figure 5:**
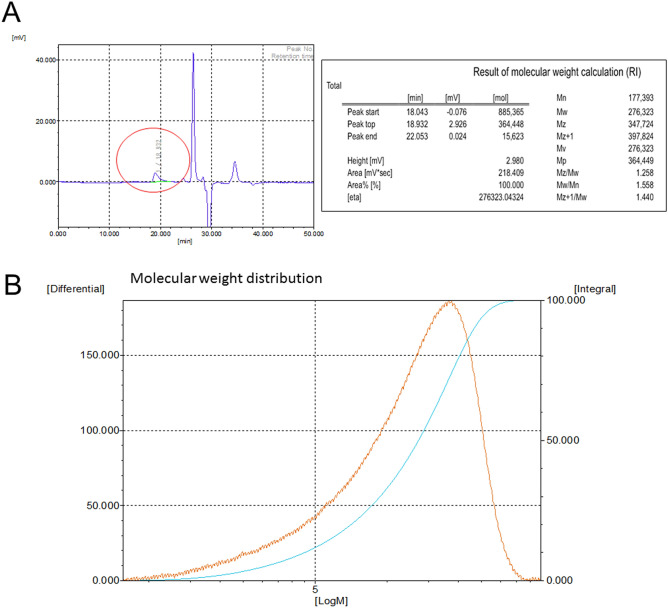
Chromatographic measurement of molecular weight released from the hyaluronic acid membrane (HAM). HAM was dissolved in normal saline at 37 °C for 48 h. The solution was analyzed with chromatography. (**A**) Hyaluronic acid peak was detected (red circle) from 18.043 min to 22.053 min. Numeral average (Mn), weight average (Mw), Z average (Mz) and polydispersity (Mw/Mn) were 177.393, 276.323, 347.724 and 1.558, respectively. (**B**) Molecular weight distribution curve of hyaluronic acid released from HAM was shown. Molecular weight of the HAM was measured using a gel permeation chromatography (Model: Tosoh EcoSec HLC-8320 GPC) with RI detector.

The HA fragments released from our HAM has average molecular weight of 276 kDa with the range of molecular weight from 15 to 885 kDa (Fig. 5).

### In vitro evaluation of HAM effect

**Figure 6:**
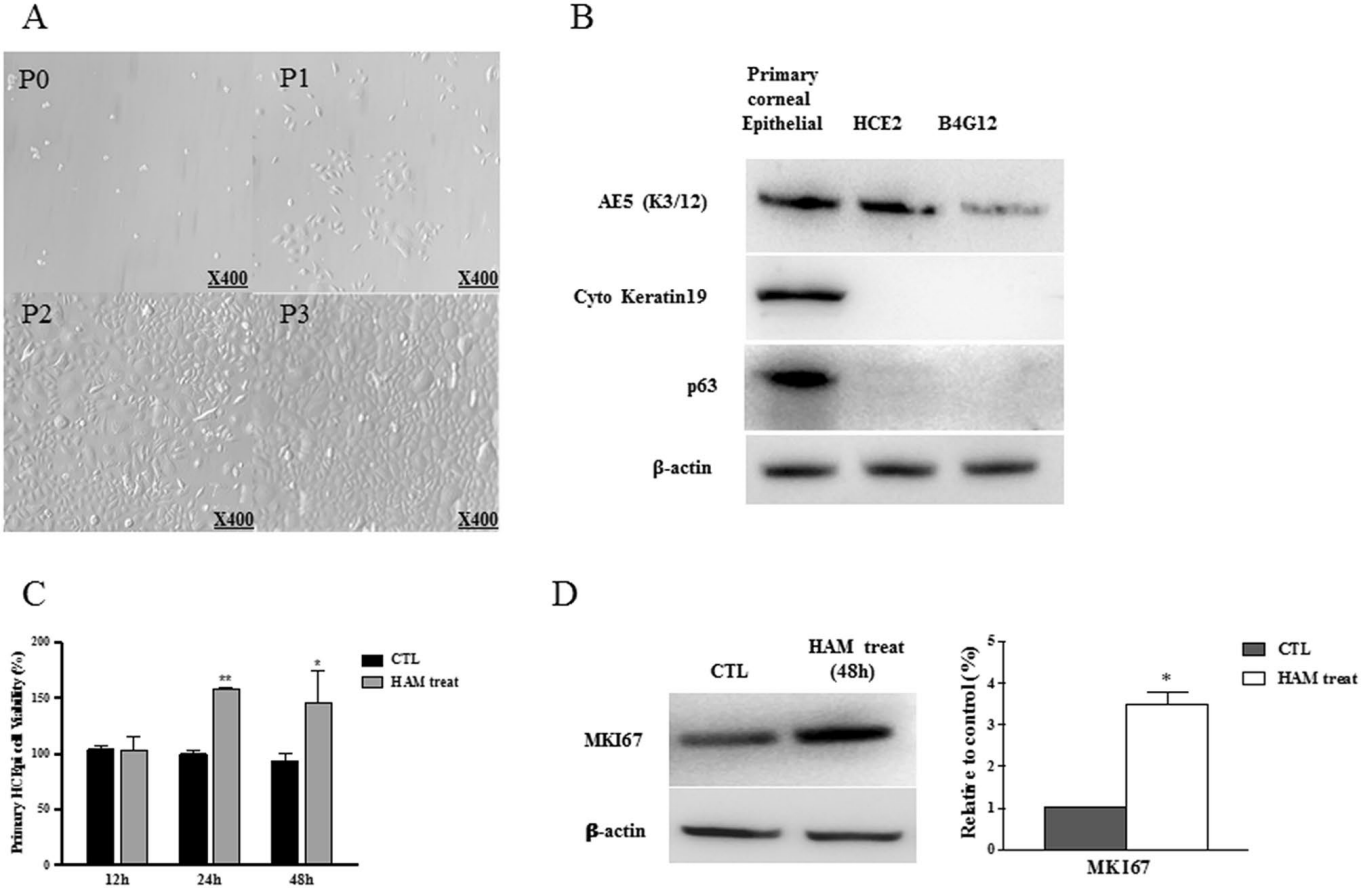
Human corneal epithelial cell culture and HAM treatment. (**A**) Phase contrast microscopic images of primary cultured human corneal epithelial cells (HCEC), Confluence of HCECs at different passages; Passages (P): P0 (day 0) P1 (day 3), P2 (day 7) P3 (day 12). (**B**) The human corneal epithelial cell phenotype was verified by the protein expression of AE5, cytokeratin 19, and p63. The human corneal epithelial cell line (HCE2, ATCC, Rockville, MD, USA) and human corneal endothelial cell line (B4G12, Creative Bioarray, Shirley, NY, USA) were used as controls. (**C**) HCEC viability was measured by CCK-8 analysis. Addition of HAM to the culture media significantly increased HCEC viability at 24 and 48 h after the culture. (**D**) MK167 is a marker for proliferation, and HAM treatment in the culture media significantly enhanced MK167 protein expression.

HCECs were successfully cultured (Fig. 6A). In order to verify the phenotype of the cultured HCECs, we investigated the expression of specific markers of primary corneal epithelial cells. The expression of K3 + 12, cytokeratin, and p63 was detected in HCECs, whereas the commercial corneal epithelial cell line and corneal endothelial cell line only expressed K3 + 12 (Fig. 6B). A subsequent cell viability test evaluated by CCK-8 analysis revealed that HAM in the culture media increased HCEC viability by 50% at 48 h (Fig. 6C). The HAM-treated cells also expressed more MK167 (also known as Ki67), which is a cellular marker for proliferation (Fig. 6D).

### In vivo evaluation of HAM effect

#### Mechanical cornea epithelial wound healing model

**Figure 7:**
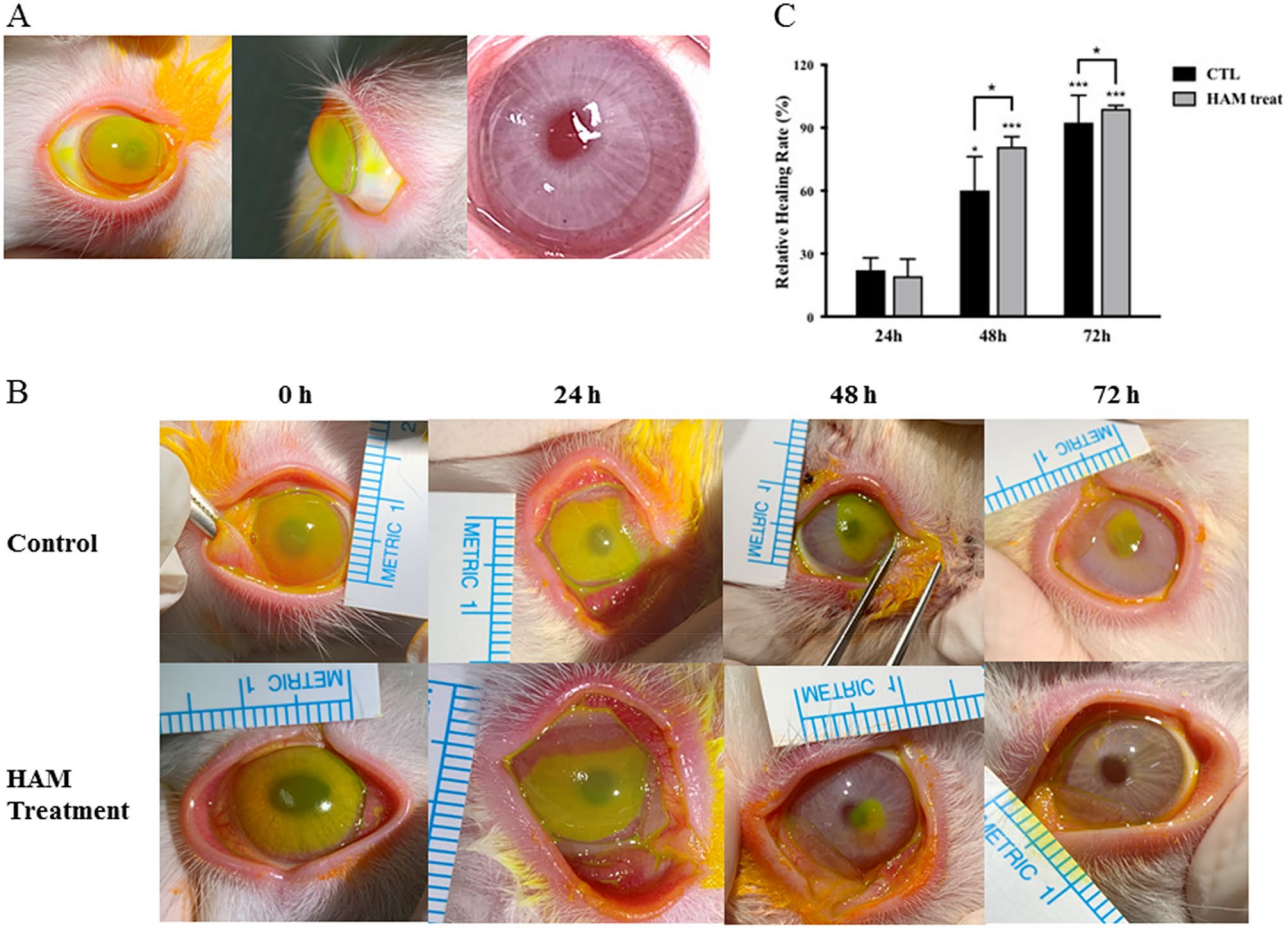
Mechanical corneal trauma model. (**A**) Representative image of HAM application on the rabbit ocular surface. The green fluorescein-stained HAM is visualized. The HAM showed excellent fit on the corneal surface. (**B**,**C**) Epithelial wound defect visualized with fluorescein staining. The green stained area represents de-epithelized cornea. As the healing occurred, the defect area decreased. HAM treatment accelerated epithelial wound healing, and a complete disappearance of the epithelial defect was observed at 72 h after the injury. However, a significant defect area was still visible in the control group at 72 h after the injury. *p < 0.05, **p < 0.01, ***p < 0.001.

When applied to the ocular surface, the HAM showed a good fit on the rabbit cornea (Fig. 7A). A mechanical corneal epithelial abrasion model was successfully created by whole corneal epithelium scraping using a no. 15 Bark Parker surgical blade on the rabbit eyes. Representative fluorescein stained images of the injured cornea are shown according to time in Fig. 7B. The area of de-epithelialized cornea was measured and compared between groups. Compared to the control group, the HAM treatment accelerated epithelial healing at 48 h. In the HAM treatment group, all corneal abrasions were completely re-epithelized at 72 h, while epithelial wound healing in the control eyes was only about 82.8% complete (p < 0.001) at 72 h (Fig. 7C).

#### Conjunctival scar model

**Figure 8:**
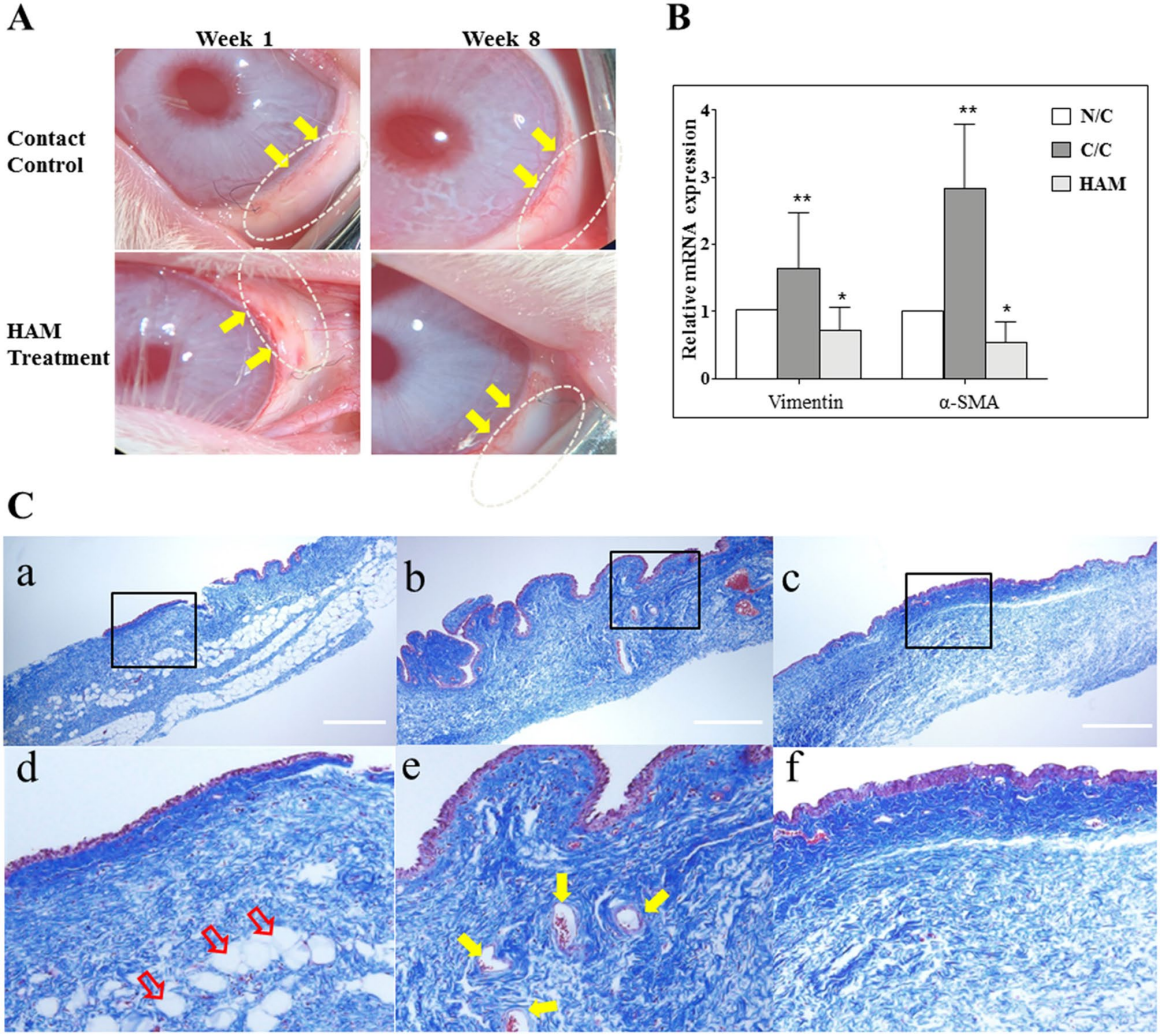
Conjunctival scar model (**A**) Surgical microscopic image. The conjunctival flap area is indicated by a white dotted oval. 10–0 nylon suture material is evident. Conjunctival mild edema and hyperemia were similar between the control and treatment groups at 1 week after the surgery. However, at 8 weeks after the surgery, the treatment groups showed superior conjunctival transparency with decreased limbal vasculatures (yellow arrows). (**B**) The gene expression for vimentin and α smooth muscle actin increased significantly in the contact control group compared to non-contact group. These fibrotic gene expressions were almost normalized by HAM treatment. *p < 0.05, **p < 0.01. (**C**) Conjunctiva were harvested and stained with Masson’s trichrome. The conjunctival epithelium is stained purple and the subconjunctival connective tissue (collagen and extracellular matrix) is stained blue. Excessive deposition of collagen and extracellular matrix, accompanied by heavy vascularization (yellow arrows), was observed in the contact control group compared with the non-contact control group. Adipose tissue (red arrows), which was normally observed in naïve conjunctiva, disappeared after the surgery. HAM treatment decreased the subconjunctival fibrosis and vascularization, but it did not restore the normal adipose tissue distribution. Panels (**d**–**f**) show the magnified images of the area indicated with rectangles in panels (**a**–**c**), respectively. The white scale bar indicates 500 μm. *NC* non-contact control, *CC* contact control, *HAM* hyaluronic acid membrane.

Simple detachment and undermining of the conjunctival flap and re-suturing can create a conjunctival adhesion and subsequent scar formation. Similar conjunctival mild edema and hyperemia induced by the surgery was observed in both the contact control and HAM treatment groups at the early postoperative period (week 1). At 8 weeks after the surgery, the HAM treatment groups showed superior conjunctival transparency and decreased limbal neovascularization compared to the contact controls (Fig. 8). The gene expression of vimentin and α SMA increased significantly in the contact controls, indicating conjunctival scar formation. However, HAM treatment almost normalized these fibrotic gene expressions. Conjunctiva harvested and stained with Masson’s trichrome showed excessive deposition of collagen and extracellular matrix, accompanied by heavy vascularization, in the conjunctival scar in the contact control group. HAM treatment decreased the subconjunctival fibrosis and vascularization to a level similar to that seen in the non-contact control group.

#### Corneal chemical burn model

**Figure 9:**
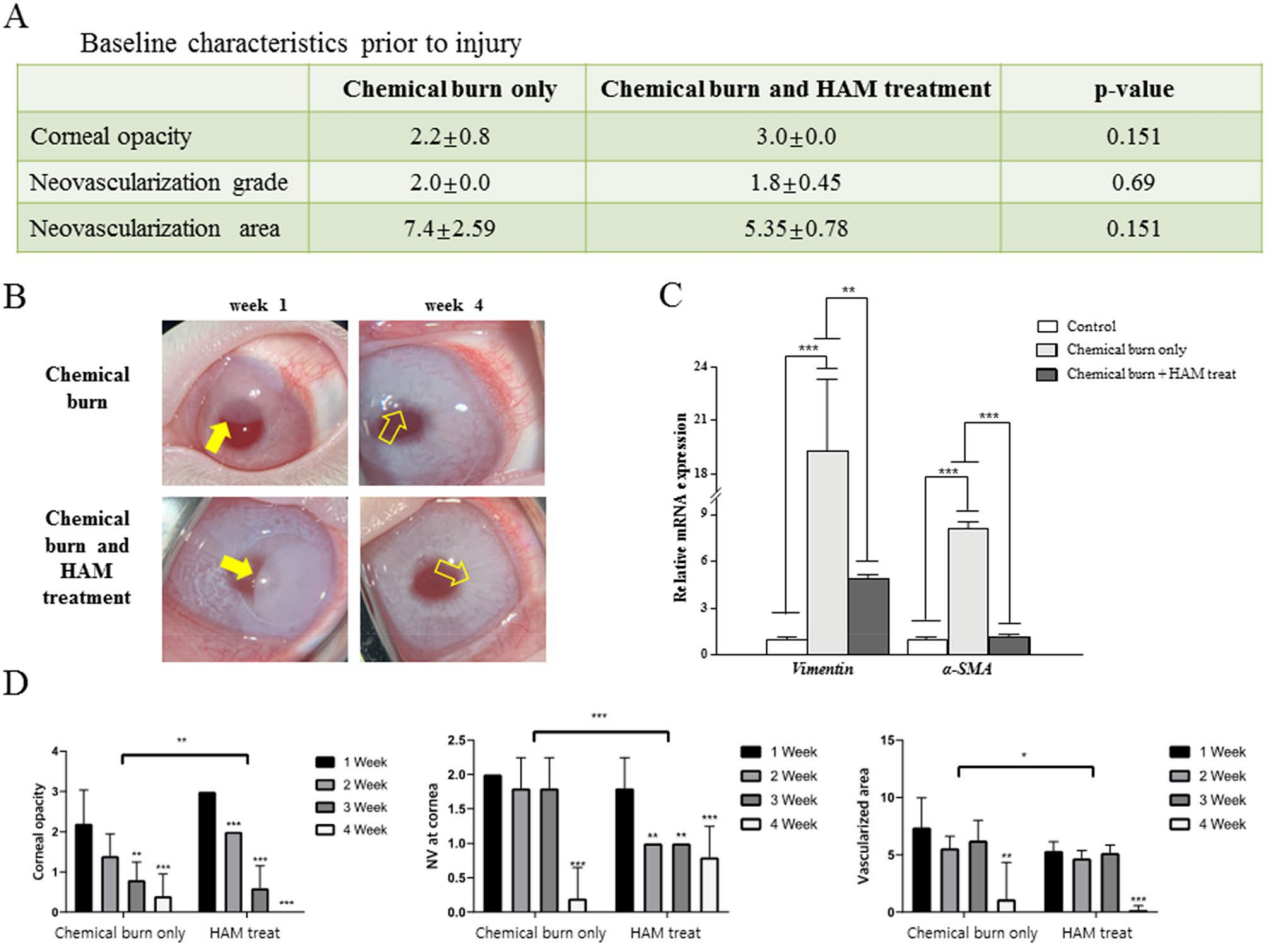
Alkali burn model. (**A**) The chemical burn only group and the HAM treatment group showed no differences in baseline characteristics in corneal opacity, neovascularization grade, and areas. (**B**) Time course of healing of corneal alkali burns. The round opaque area (yellow arrows) indicates a successful alkaline burn on the corneas (week 1). At 4 weeks after the injury, corneal transparency was grossly recovered in both the chemical burn only group and HAM treatment group (blank arrows). (**C**) Gene expression of vimentin and α smooth muscle actin increased significantly in the chemical burn group compared to the control (naïve cornea) group. However treatment with HAM significantly blunted the vimentin expression and almost normalized the α smooth muscle actin expression. **p < 0.01, ***p < 0.001. (**D**) Both the chemical burn only and HAM treatment groups showed significant improvement in corneal opacity, neovascularization grade, and neovascularization area as healing progressed for 4 weeks. However, the HAM treatment achieved a better corneal transparency and neovascularization area at 4 weeks and a more rapid improvement of neovascularization grade at 3 weeks. Comparisons of the healing of chemical burns in HAM-treated versus control were performed by using a 2-way repeated measured analysis of variance (ANOVA). *p < 0.05, **p < 0.01, ***p < 0.001.

Alkali burns of the cornea are one of the most severe corneal injuries and develop serious complications, such as corneal opacity and neovascularization. The baseline neovascularization and corneal opacity grade was measured prior to the alkali burn. To confirm the efficacy of the HAM, the final state of the corneal opacity and neovascularization was compared to the baseline measurement (Fig. 9). An alkali burn resulted in significant cornea edema and opacity at 1 week after the injury. However, at 4 weeks after the injury, corneal transparency had improved in both the chemical burn only group and the HAM treatment group. The HAM treatment showed better corneal transparency and an improved neovascularization area at 4 weeks, as well as a more rapid improvement in the neovascularization grade at 2 and 3 weeks. The gene expression of vimentin and α-SMA increased significantly after the chemical burn. However, HAM treatment significantly blunted this vimentin expression and almost normalized α smooth muscle actin expression.

## Discussion

In this study, we verified the safety and efficacy of HAM for the treatment of various OSDs. Several reports have shown that cross-linked polymer eye drops or membrane platforms could be useful treatment modalities in clinical ophthalmology^35–37^. However, our study is the first to demonstrate that a novel HAM, produced using a BDDE cross-linker, is a potentially valid treatment for several OSDs, with promising results both in vitro and in vivo.

The strength of polymer cross-linking is determined by the concentration of the cross-linker^33,38^. BDDE is recognized as a low-toxicity cross-linker, so it is widely used as a chemical cross linker in various biological research fields. However, it has potential toxicity depending on the type of cell and the exposure concentration^39,40^. Determining the optimal BDDE concentration for cross-linking is therefore an important step for HAM manufacture. In this study, we found that primary cultured HCECs could be safely exposed for 48 h to BDDE at concentrations less than 0.01%. Based on these data, the HAM was manufactured by mixing equal volumes of 0.02% BDDE and HA solution to give a final BDDE concentration of 0.01%. As expected, the HAM was well tolerated both in vitro in a corneal epithelial cell culture and in vivo in rabbit eyes. For further verification, measurement of the residual BDDE in the HAM by GC–MS revealed concentrations less than 0.05 ppm (this is the limit of lowest detection concentration in our experiment).

In the dry state, the HAM had a thin and firm membrane property. Therefore, designing the required shape of HAM by the surgeon will be easy and can provide the best fit for the surface on which it will be applied. When hydrated, the HAM is also soft and flexible so it can fit well on curved surfaces, like a soft contact lens. The transparency of the HAM is superior, with an optical transmittance of over 91% for visible light even in the hydrated state. This high transparency will help physicians when examining the healing process of the ocular surface.

The structure and chemical modification of the HAM were also analyzed by SEM and FT-IR. SEM images of the HAM revealed a homogenous and rigid structure with a high density of small pores ranging in size from a few microns to around 75 µm. The morphological structure and interconnectivity is known to depend on the strength of the cross-linking^41^. The new chemical bonds in the HAM induced by cross-linking were confirmed by FT-IR. As shown in Fig. 3, the chemical modification of HAM was evident by the difference in spectra between the HAM and the native HA. One interesting finding is a small peak at 2900 cm^−1^ in the HAM. Because this peak represented C-H stretching in the cross-linker, the HAM shows that peak but the native HA does not. Under alkaline conditions, the epoxide group of BDDE binds with the hydroxyl groups of HA and forms an ether bond. This ether bond can be verified by the peak 2 appearing at around 1300 cm^−1^ in the FT-IR spectrum.

HA is normally distributed in various tissue fluids in the human body. Although cartilage, skin and synovial fluid contain mostly high molecular with HA (> 1000 kDa), HAs found in saliva, blood and amniotic fluid are the mixture of high and low molecular weight HAs^42^. Especially, ocular tissues are different from other tissues in that there are no blood vessels. Consequently the average molecular weight of HA in ocular tissues is very high^42^*.* Previous studies suggested that HA could have different roles in wound healing process depending on the size of HA fragments. High molecular weight HA (> 1000 kDa) was reported to have some immunosuppressive, anti-inflammatory and anti-angiogenic effect, whereas low molecular weight HA, such as oligomers of 8–16 disaccharides, may stimulate inflammatory response and tissue angiogenesis^9–11^. However, this size dependent HA effect can be different according to cell types and tissue^43^. Although, a further investigation is needed, there is a possibility that wound healing modulated by HAM application may be due to the combined effect of both high and low molecular weight of HA.

The safety of the HAM was investigated using primary culture of HCECs. As shown in Fig. 6, cell viability increased when the HAM was added in the culture medium. The enhanced proliferation of HCECs was further verified by the increased expression of MK167, which is a marker for cell proliferation^6^. This finding suggests a potential of the HAM to enhance corneal epithelial wound healing.

The efficacy of the HAM in OSD was verified using three different disease models. In mechanical corneal epithelial injury model, HAM treatment enhanced epithelial wound healing, as demonstrated. The retention of the HAM on the rabbit cornea was monitored on a daily basis and evaluated by visual observation of the corneal appearance. The HAM maintained its membranous form for 48 h after application to the ocular surface. After 48 h, the membrane changed to a gel, but it still covered the ocular surface. This finding suggests that the HAM can function as a bioactive extracellular matrix (ECM) graft, similar to amniotic membrane. As is well known, HA is one of the abundant ECM macromolecules and works as a mechanical support for various tissues^35,44^. In addition, HA enhances corneal and conjunctival wound healing via CD44. We believe that three-dimensional scaffolds of a HAM may enable this mechanical and biological function.

The development of fibrosis and adhesion is a common complication after conjunctival surgeries, such as pterygium removal or glaucoma filtration surgery. This fibrosis provokes ocular discomfort and filtration failure of trabeculectomy. Several studies have shown that hyaluronic acid effectively prevents post-operative fibrosis in several clinical fields and even in animal studies^45–47^. This anti-fibrotic effect is known to be a physical barrier between separated tissues during early healing process and reduces and delays postoperative scar formation. Consistent with previous studies, our results demonstrated that the HAM had anti-fibrotic effect in the conjunctival scar model. Excessive subepithelial accumulation of collagen and ECM was observed at the contact control group, but the HAM- treated conjunctiva showed a normalized histology. We further examined the level of fibrosis at the surgical wounds by measuring the expressions of vimentin and α-SMA^45,48–50^, which are responsible for induction of scar formation and wound contraction^51^. As show in Fig. 8, HAM treatment attenuated the expression level of α-SMA and vimentin compared to the control eyes.

An alkali burn on the ocular surface usually induces a full penetrating injury of the cornea from the epithelium to the endothelium through the stroma. Therefore, timely and adequate treatment is important to prevent corneal blindness by opacities and neovascularization secondary to the alkali burn. Our use of the corneal alkali burn model allowed evaluation of the efficacy and safety of the HAM. When observed at 4 weeks after the injury, corneal opacity decreased from 3 to 0 in the HAM treatment group, whereas corneal opacity only decreased from 2.2 to 0.4 in the control group, suggesting a faster restoration of corneal transparency using HAM. Corneal neovascularization was also significantly decreased in the HAM treatment group.

Alkali burns can activate keratocytes to differentiate into myofibroblasts and result in corneal opacity. Timely re-epithelization is known to be critical in preventing myofibroblast formation in the cornea^52^. As previously discussed, HAM treatment could promote corneal epithelial wound healing and reduce the expression of fibrotic markers. Corneal neovascularization is another complication of corneal injury, and neovascularized corneas are vulnerable to inflammatory reaction. Chronic inflammation further aggravates corneal opacity, so the finding that HAM treatment decreased corneal neovascularization also indicated a promising role of HAM. Overall, these results demonstrated that HAM treatment could obtain faster restoration of corneal transparency with reduced neovascularization when applied to corneal alkali burns.

Chemical crosslinking of HA for ophthalmic use was attempted previously by other researchers^23,53^. Thiolated carboxymethyl HA has been crosslinked using poly (ethylene glycol) diacrylate to make a gel or an opaque membrane (CMHA-S) for potential ophthalmic use. The effect of crosslinked HA gel has also verified in various animal models. Our HAM has distinct characteristics compared to CMHA-S. We used non-chemically modified HA and BDDE as a crosslinker. The resulting HAM is transparent both in the dry and hydrated states. The CMHA-S membrane is opaque and flexible in the dry state, whereas the HAM is transparent and firm when dry.

Our study has several limitations. We verified the safety of our HAM using HCECs; however, cultured cells may not fully represent the typical characteristics of the corneal epithelium in living humans. For instance, the normal delicate interaction with various tear components and the eyelid could not be reproduced in an in vitro culture environment. Although we tested the efficacy in three OSDs animal models, the wound healing responses of rabbits might differ from those in humans, so the results might not completely represent the human responses. Another limitation is the inevitable use of animals for efficacy evaluations. The original purpose of the HAM was to apply it to the human ocular surface as it was anticipated to maintain its form for several days to modulate wound healing. However, several consecutive HAM applications were needed at each observation point due to the uncontrollable blinking and rubbing behavior by the rabbits.

In summary, we demonstrated the safety and efficacy of a HAM. Our HAM showed excellent physical properties, such as easy manipulation, excellent transparency, and good fitting on the ocular surface. We used various OSD models to successfully demonstrate the efficacy of using a HAM in the treatment of several OSDs. HAM application could modulate the wound healing response and result in the favorable OSD outcomes.

## Materials and methods

### Preparation of HAM

An initial cell viability test using human corneal epithelial cells (HCECs) was conducted to determine the acceptable BDDE concentration in the HAM. The cells were seeded, incubated for 24 h, and then exposed to various concentrations of BDDE (0.5, 1, 2.5, 3, and 5%; Sigma Aldrich, St. Louis, MO, USA). The cell viability was then assessed using CCK-8 reagent (Dojindo Molecular Technologies, Inc., Kumamoto, Japan), according to the manufacturer’s protocol.

**Figure 10:**
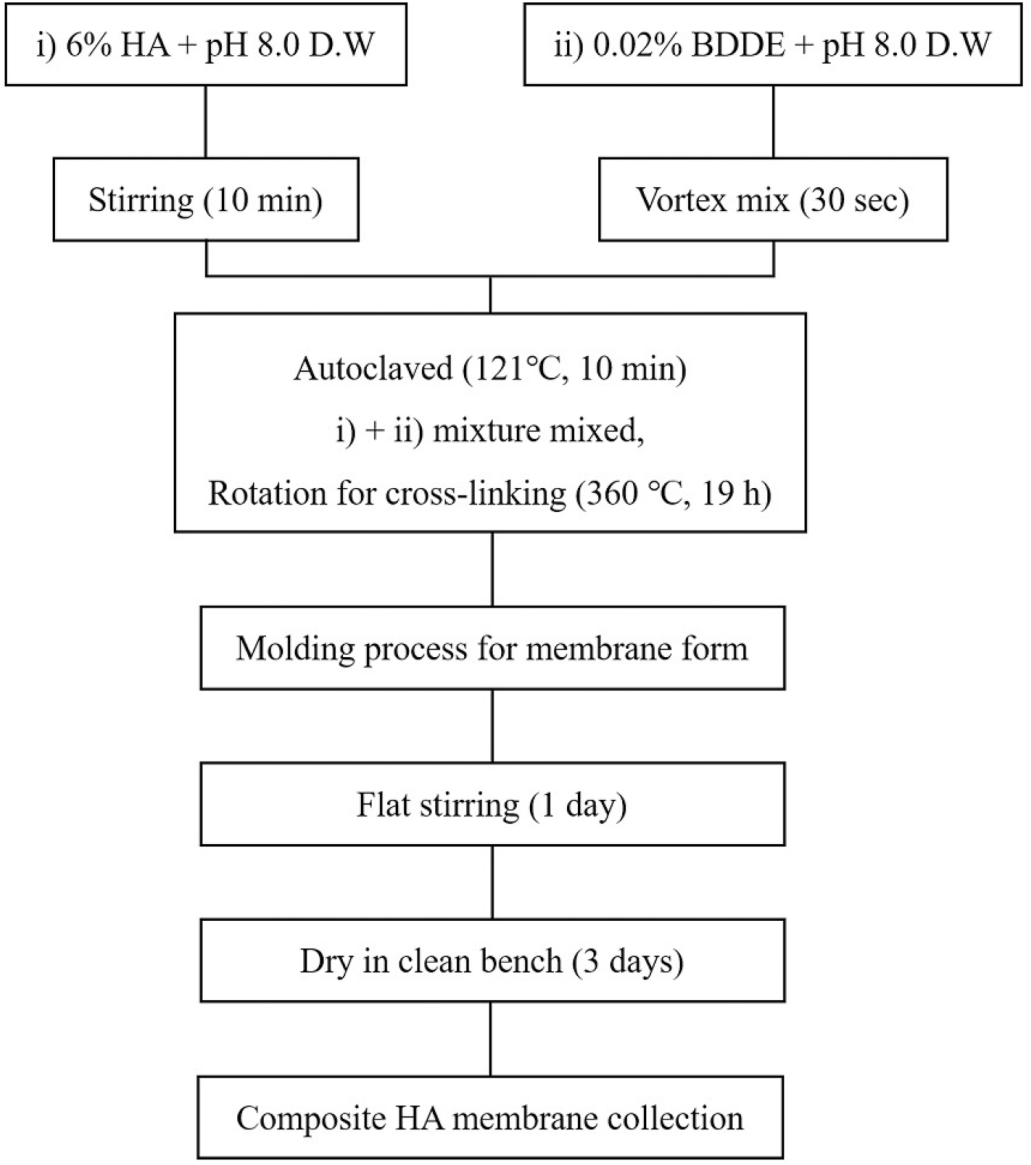
A schematic diagram of hyaluronic acid membrane (HAM) manufacture.

HAM was manufactured by crosslinking 6% HA (average molecular weight = 700,000 Da, SK Bioland, Cheonan, South Korea) with 0.01% BDDE. The mixture was incubated at room temperature (RT) for 19 h and then spread onto a plate for membrane formation (24 h of flat stirring and 72 h of drying on a clean bench at RT). Details of the manufacturing procedure are shown in Fig. 10. Every step was carried out under aseptic conditions.

### Physical characterization of HAM

#### Swelling ratio and water absorption capacity

Dry HAM was cut into circles of 5 mm diameter. All samples were hydrated in normal saline until they reached an equilibrium state. The remaining water was blotted with absorption paper. The swelling ratio of the HAM was determined by measuring the diameters of the circles after 5 days.

The water absorption capacity of the HAM was determined by comparing the water content before and after the hydration. The water content in the HAM was measured by the well-established Karl-Fischer titration technique^54^. The dry HAM was evaluated by a coulometric method (Mettler Toledo, Metrohm 852 Titrando Karl Fisher Titrator with Metrohm 874 Oven Sample Processor, Germany), and the fully hydrated HAM was evaluated by a volumetric method (Mettler Toledo, Metrohm 852 Titrando Karl Fisher Titrator Metrohm 860 KF Thermoprep, Germany).

#### Fourier-transformed infrared (FT-IR) analysis

The chemical compositions of native and cross-linked hyaluronic acid were determined by Fourier transform infrared spectroscopy FTIR (FT/IR-4600, Jasco, Oklahoma City, OK, USA). We measured the transmittance of the samples in the Attenuated total reflection (ATR) mode. Spectra were obtained with 32 scans, with a resolution of 4 cm^−1^ and a range of wavenumbers from 4000 cm^−1^ to 500 cm^−1^. The Spectra Manager software (Jasco, Oklahoma City, OK, USA) was used to analyze the data.

#### Scanning electron microscope (SEM)

The morphologies of native and cross-linked hyaluronic acid were visualized by scanning electron microscopy (SEM) using an IT-500HR instrument (JEOL, Tokyo, Japan). All images were obtained using the Secondary Electron Imaging (SEI) mode with a voltage of 15 kV. The samples were prepared by freezing the hydrogel solution overnight and then soaking in liquid nitrogen for 1 h. The sample was fixed onto an aluminum plate with carbon tape and sputter coated with platinum. The pore size was measured using image analysis software (ImageJ, Scion Corp., Frederick, MD, USA).

#### Measurement of optical transmittance

The transmittance spectra of the hydrated and dry HAM were measured using a Scinco UV–Vis scanning spectrometer (Model S-4100, Scinco, Daejeon, South Korea). The HAM was hydrated in normal saline for 5 min and each HAM was cut with a punch into circles with a diameter of 5 mm. The optical transmittance of the HAM was measured at visible light. The transparency of the HAM was analyzed by UV–Vis analysis.

#### Gas chromatography-mass spectrometry (GC–MS) analysis for detecting residual BDDE

The BDDE was eluted using two types of solvent: methanol and saline. Approximately 10 mg of HAM was eluted in 1 g of methanol for 1 h or in saline for 3 h and 24 h. A reference solution of 2 ppm BDDE was prepared in saline. The removal of BDDE residue from the HAM was tested by gas chromatography–mass spectrometry (GC–MS) analysis (7890B-5977A, Agilent Technologies, CA, USA).

#### Measurement of molecular weight of HA released from HAM by hydrolysis

Chromatographic measurement of molecular weight released from the hyaluronic acid membrane (HAM). HAM was dissolved in normal saline at 37 °C for 48 h. The solution was analyzed with chromatography (Model: Tosoh EcoSec HLC-8320 GPC) with RI detector. GPC equipment had connected columns (2 × TSKgel GMPW_XL_ + TSKgel G2500PW_XL_) and columns were kept at 40 °C. The eluent was 0.1 M NaNO3. The flow rate was 1 mL/min. The result from GPC system was analyzed using EcoSec software.

### In vitro evaluation of HAM effect

#### Primary human corneal epithelial cell culture

Primary culture of human corneal epithelial cells (HCECs) was performed using a cadaveric donor corneal tissue not suitable for clinical use (Eversight Korea, Seoul, South Korea). The HCECs were cultured in corneal epithelial cell basal medium (PCS-700-030, ATCC, USA) supplemented with a corneal epithelial cell growth kit (PCS-700-040, ATCC, USA) at 37 °C in a humidified 5% CO_2_ atmosphere. The anterior lamella was cut into explant cubes of approximately 2 × 2 mm with a scalpel under a microscope and aseptic conditions. Six to eight explants were placed epithelium side down on a 24-well plate coated with a fibronectin and collagen (FNC) coating mix (AthenaES, Baltimore, MD, USA) and containing growth medium. The explants were cultured at 37 °C overnight and then removed from the wells. The adherent corneal epithelial cells were maintained by adding medium every two days. When the cells reached 80% confluence, they were passaged at a 1:3 ratio. Cells from passages 3 to 4 were used for the experiments.

The expression of corneal epithelial cell markers AE5 and cytokeratin 19 and p63 was analyzed by western blotting. Beta-actin was used as a loading control. Immortalized commercial human corneal epithelial cells (HCE2, ATCC, Rockville, MD, USA) and human corneal endothelial cells (B4G12, Creative Bioarray, Shirley, NY, USA) were used as controls.

#### Human corneal epithelial cell viability assay

We assessed cell viability using CCK-8 reagent (Dojindo Molecular Technologies, Inc., Kumamoto, Japan), according to the manufacturer’s protocol. Briefly, primary HCECs were cultured at 4 × 10^3^ cells/well in a 96-well plate, incubated for 24 h, and then the HAM was applied to the adhered cells. Cells were incubated with the HAM and without the HAM for 12, 24, and 48 h. CCK-8 solution was added to each well and the plates were incubated for 1–4 h. Viable cells were detected by determining the absorbance values at 450 nm using a microplate reader.

### In vivo evaluation of HAM effect

#### Animals

Male specific-pathogen-free (SPF) rabbits (n = 30, 2.0–2.5 kg) were purchased from the Kangda Rabbit industry (Qingdao, China). The sample size was estimated using a power analysis (SigmaPlot). Ten rabbits (n = 10) were used for a mechanical cornea trauma study (group 1 and 2) and another 10 rabbits were used for a conjunctival wound healing study (group 3 and 4). The remaining rabbits were used for a chemical burn study (group 5 and 6). Animals were treated in compliance with the ARVO Statement for the Use of Animals in Ophthalmic and Vision Research and the ARRIVE (Animal Research Reporting of In Vivo Experiments) guidelines. The experimental protocol was approved by the Institutional Animal Care and Use Committee (IACUC) of Dongguk University, Ilsan Hospital (reference number: 2016-03146).

#### Mechanical cornea epithelial wound model

After systemic Zoletil (Virbac Corporation, Fort Worth, TX, USA) and topical Alcaine (Alcon Laboratory, Fort Worth, TX, USA) anesthesia, the whole corneal epithelium of each rabbit’s right eye was removed by scraping using no.15 Bard Parker blade. The epithelial defect was verified by fluorescein staining. Five rabbits (group 1) received a HAM on the cornea surface and the lids were suture-closed using 5-0 black silk. The other five rabbits (group 2) were used as a control and the lids were suture closed without HAM application. The HAMs were fully hydrated by soaking in PBS just before application and settling onto the cornea surface was confirmed before lid closure. After 24 h, the sutures were removed and the lids were opened. Corneal epithelial healing was monitored by examination under fluorescein staining at 24, 48, and 72 h. During the examination, stained images were obtained and the wound area was measured using image analysis software (ImageJ, Scion Corp., Frederick, MD, USA). Because the remnant of the HAM was inevitably removed during the topical anesthesia and washing procedure, another set of HAMs was inserted in the group 1 rabbits at each examination (24 h and 48 h) and lids were sutured closed. The group 2 rabbits underwent lid closure only, without HAM application, at each examination. We also checked the HAM to determine when it disappeared in vivo.

#### Conjunctival scar model

After systemic Zoletil and topical Alcaine anesthesia, a superior conjunctival limbal periotomy was performed about from 10 to 2 o’clock meridian in the right eye of each rabbit. Blunt dissection was performed to ensure undermining of a 10 × 10 mm sized section of the conjunctiva. Five rabbits (group 3) received HAM in the sub-conjunctival space of the dissected area and conjunctiva was sutured to the limbus using 10-0 nylon. The other five rabbits (group 4) were used as a control, and the conjunctiva was sutured to the limbus with no HAM insertion. The control group was further divided into two sub-groups: (a) a contact control (with undermined conjunctiva without HAM insertion), and (b) a non-contact control (left eyes, without manipulated conjunctiva). Rabbits were examined at week 1 and 2 and then every 2 weeks until 2 months. Conjunctival hyperemia was monitored. After 2 months, the rabbits were euthanized and tissues were harvested.

#### Alkali burn model

After systemic Zoletil and topical Alcaine anesthesia, corneal alkali burns were created on the superocentral cornea for 120 s using a circular 6.0 mm filter paper fully soaked in 0.1 N NaOH. Each burned cornea was then rinsed with balanced salt solution (BSS) for 2 min. The HAM treated group (group 5) had a HAM applied to the burn area once per week for 3 weeks. The chemical burn only group (group 6) had nothing applied to the burn area. Antibiotic eye drops (0.5% levofloxacin, Cravit, Santen, Osaka, Japan) were also applied topically in both groups after the chemical burn. The efficacy of HAM was evaluated based on corneal opacity and neovascularization determined by photographing the cornea with a slit lamp with a digital camera (iPhone) at 0, 1, 2, and 4 weeks. Corneal opacity assessment followed the Roper-Hall classification^55^ and was scored on a scale of 0–4, where 0 = completely clear; 1 = slightly hazy, but iris details visible; 2 = stromal haze but iris detail visible, 3 = stromal haze obscures iris details, 4 = cornea opaque with no view of iris. Corneal neovascularization assessment followed the protocol of a previous study^56^. The neovascularization was scored on a scale of 0–4, where 0 = no vessels at the corneal limbus; 1 = vessels within 1 mm of the corneal limbus; 2 = vessels within 2 mm of the corneal limbus; 3 = vessels 4 mm over the corneal limbus to the corneal center, and 4 = vessels within 2 mm of the corneal center. The vascularized area was determined from photographs using image J.

#### Histology

Rabbit eyes were histopathologically studied to examine the effect of the HAM. The rabbits were sacrificed at 2 months after the injury. Conjunctival sections (10 × 10 mm in size) were removed from the previous wound site at 2 months after the wounding from the rabbits in group 3 and 4. The conjunctiva of the left eyes of group 4 was used as non-contact controls. The tissues were divided in half, with each piece containing the limbal conjunctiva. One half of the tissue was fixed with 10% formalin and embedded in paraffin, sectioned (4 μm), and stained with Masson’s Trichrome. The other half was used for PCR analysis to examine the proliferative activity of the cells.

#### Total RNA extraction and single-strand cDNA synthesis

After exposure to the HAM, primary corneal epithelial cells were harvested, ground up with a grinder in 5 volumes of TRIZOL reagent (Molecular Research Center, Inc.), and stored at 80 °C until use. Total RNA was isolated from harvested primary corneal epithelial cells according to the manufacturer’s instructions. Total RNA quality and quantity was confirmed using 1% agarose gels with RedSafe (Intron Biotechnology) staining, a UV transilluminator (Vilber Lourmat, TFX-20), and a Nano drop spectrophotometer (ACTGene Inc., ASP-2680). Single-strand cDNA was synthesized from 1 μg of total RNA using random primers for reverse transcription (Toyobo, ReverTra Ace qPCR RT Master Mix, Japan).

#### Quantitative real-time reverse transcriptase polymerase chain reaction (real-time RT-PCR)

The selected primer sets are shown in Table 1. The expression patterns of rabbit (*Oryctolagus cuniculus*) genes were determined by quantitative real-time RT-PCR. Each PCR reaction included 1 μl of cDNA and 0.2 μM of a primer set. The reaction conditions were as follows: 95 °C/5 min; 45 cycles of 95 °C/10 s, 55 °C/10 s, 72 °C/10 s; and 72 °C/10 min. To verify that each primer only hybridized to the target sequence, RT-PCR was performed using LightCycler 480 (Roche) and the PCR products were analyzed on a 1.5% agarose gel. Only a single band was visualized on the gel by UV exposure. The amplification of the specific product was confirmed by continuing the cycles to allow checking of the melting curve at the following conditions: 95 °C/1 min, 55 °C/1 min and 80 cycles of 55 °C/10 s with a 0.5 °C increase per cycle. LightCycler 480 SYBR Green (Roche) was used to detect the specific amplified products. Amplification and detection of the SYBR Green-labeled products were performed using IDEAS 2.0 (Roche, USA). Data from each experiment were expressed as relative expression levels of the rabbit GAPDH gene to normalize the expression levels between samples, as suggested by Small et al.^57^. All experiments were conducted in triplicate. Data were collected as threshold cycle (CT) values (PCR cycle number, where fluorescence was detected above a threshold and decreased linearly with increasing input target quantity) and used to calculate the ΔCT values of each sample. The fold change in the relative gene expression was calculated by the 2-ΔΔCT method^58^.

**Table 1:** Primers used for the polymerase chain reaction.

Species	Gene	Accession number		Primer sequence (5′-3′)	Length (base pair)
Rabbit	Vimentin	XM_002717420	F	AGATGCGTGAAATGGAAGAG	296
			R	TGTCCTTTTTGAGTGGGTGT	
Rabbit	⍺-Smooth muscle actin	NM_001101682	F	TGCTGTCCCTCTATGCCTCT	185
			R	GAAGGAATAGCCACGCTCAG	
Rabbit	GAPDH	NM_001082253	F	TGACGACATCAAGAAGGTGGTG	121
			R	GAAGGTAGAGGAGTGGGTGGC	

*GAPDH* glyceraldehyde 3-phosphate dehydrogenase, *F* forward, *R* reverse.

### Statistics

Data are presented as mean ± standard error, and statistical significance was determined using a one-way ANOVA, followed by the Dunnett’s multiple comparison test. The chemical burn data were evaluated by repeated measured ANOVA, followed by Bonferroni’s method. In this study, statistical significance was expressed as asterisks, using NEJM formatting for P values, and calculations were made using GraphPad Prism Ver. 7.01 (GraphPad Software, Inc., La Jolla, CA, USA).

## Supplementary Information

Supplementary Information

## Data Availability

The datasets generated during and/or analysed during the current study are available from the corresponding author on reasonable request.

## References

[1] Gipson IK (2007). The ocular surface: The challenge to enable and protect vision: The Friedenwald lecture. Invest. Ophthalmol. Vis. Sci..

[2] Ang BCH, Sng JJ, Wang PXH, Htoon HM, Tong LHT (2017). Sodium hyaluronate in the treatment of dry eye syndrome: A systematic review and meta-analysis. Sci. Rep..

[3] Price RD, Myers S, Leigh IM, Navsaria HA (2005). The role of hyaluronic acid in wound healing: Assessment of clinical evidence. Am. J. Clin. Dermatol..

[4] Carlson E, Kao WWY, Ogundele A (2018). Impact of hyaluronic acid-containing artificial tear products on reepithelialization in an in vivo corneal wound model. J. Ocul. Pharmacol. Ther..

[5] Presti D, Scott JE (1994). Hyaluronan-mediated protective effect against cell damage caused by enzymatically produced hydroxyl (OH·) radicals is dependent on hyaluronan molecular mass. Cell Biochem. Funct..

[6] Gomes JA, Amankwah R, Powell-Richards A, Dua HS (2004). Sodium hyaluronate (hyaluronic acid) promotes migration of human corneal epithelial cells in vitro. Br. J. Ophthalmol..

[7] Wu CL (2013). Hyaluronic acid-dependent protection against alkali-burned human corneal cells. Electrophoresis.

[8] Mero A, Campisi M (2014). Hyaluronic acid bioconjugates for the delivery of bioactive molecules. Polymers (Basel).

[9] Aya KL, Stern R (2014). Hyaluronan in wound healing: Rediscovering a major player. Wound Repair Regen..

[10] Jiang D, Liang J, Noble PW (2007). Hyaluronan in tissue injury and repair. Annu. Rev. Cell Dev. Biol..

[11] Litwiniuk M, Krejner A, Speyrer MS, Gauto AR, Grzela T (2016). Hyaluronic acid in inflammation and tissue regeneration. Wounds.

[12] Aragona P, Papa V, Micali A, Santocono M, Milazzo G (2002). Long term treatment with sodium hyaluronate-containing artificial tears reduces ocular surface damage in patients with dry eye. Br. J. Ophthalmol..

[13] Entwistle J, Hall CL, Turley EA (1996). HA receptors: Regulators of signalling to the cytoskeleton. J. Cell Biochem..

[14] Zhu SN, Nolle B, Duncker G (1997). Expression of adhesion molecule CD44 on human corneas. Br. J. Ophthalmol..

[15] Lerner LE, Schwartz DM, Hwang DG, Howes EL, Stern R (1998). Hyaluronan and CD44 in the human cornea and limbal conjunctiva. Exp. Eye Res..

[16] Yu FX, Guo J, Zhang Q (1998). Expression and distribution of adhesion molecule CD44 in healing corneal epithelia. Invest. Ophthalmol. Vis. Sci..

[17] Iocono JA, Ehrlich HP, Keefer KA, Krummel TM (1998). Hyaluronan induces scarless repair in mouse limb organ culture. J. Pediatr. Surg..

[18] Mast BA, Diegelmann RF, Krummel TM, Cohen IK (1993). Hyaluronic acid modulates proliferation, collagen and protein synthesis of cultured fetal fibroblasts. Matrix.

[19] Benedetti L (1993). Biocompatibility and biodegradation of different hyaluronan derivatives (Hyaff) implanted in rats. Biomaterials.

[20] Johnson ME, Murphy PJ, Boulton M (2006). Effectiveness of sodium hyaluronate eyedrops in the treatment of dry eye. Graefes Arch. Clin. Exp. Ophthalmol..

[21] Durrani AM, Farr SJ, Kellaway IW (1995). Influence of molecular weight and formulation pH on the precorneal clearance rate of hyaluronic acid in the rabbit eye. Int. J. Pharm..

[22] Yavuz B, Kompella UB (2017). Ocular drug delivery. Handb. Exp. Pharmacol..

[23] Griffith GL (2018). Treatment of corneal chemical alkali burns with a crosslinked thiolated hyaluronic acid film. Burns.

[24] Meller D (2000). Amniotic membrane transplantation for acute chemical or thermal burns. Ophthalmology.

[25] Baum J (2002). Thygeson lecture. Amniotic membrane transplantation: Why is it effective?. Cornea.

[26] Williams DL, Mann BK (2013). A crosslinked HA-based hydrogel ameliorates dry eye symptoms in dogs. Int. J. Biomater..

[27] Tredici C, Fasciani R, Villano A, Gambini G, Caporossi A (2020). Efficacy of eye drops containing crosslinked hyaluronic acid and CoQ10 in restoring ocular health exposed to chlorinated water. Eur. J. Ophthalmol..

[28] Fallacara A, Manfredini S, Durini E, Vertuani S (2017). Hyaluronic acid fillers in soft tissue regeneration. Facial Plast. Surg..

[29] Valachova K, Volpi N, Stern R, Soltes L (2016). Hyaluronan in medical practice. Curr. Med. Chem..

[30] Yang G, Espandar L, Mamalis N, Prestwich GD (2010). A cross-linked hyaluronan gel accelerates healing of corneal epithelial abrasion and alkali burn injuries in rabbits. Vet. Ophthalmol..

[31] Schanté CE, Zuber G, Herlin C, Vandamme TF (2011). Chemical modifications of hyaluronic acid for the synthesis of derivatives for a broad range of biomedical applications. Carbohyd. Polym..

[32] Zhao X (2006). Synthesis and characterization of a novel hyaluronic acid hydrogel. J. Biomater. Sci. Polym. Ed..

[33] Al-Sibani M, Al-Harrasi A, Neubert RH (2016). Study of the effect of mixing approach on cross-linking efficiency of hyaluronic acid-based hydrogel cross-linked with 1,4-butanediol diglycidyl ether. Eur. J. Pharm. Sci..

[34] Al-Sibani M, Neubert Rhh AA-H (2018). Characterization of linear and chemically cross-linked hyaluronic acid using various analytical techniques including FTIR, ESI-MS, H1 NMR, and SEM. J. Biochem. Anal. Stud..

[35] Kim H (2017). Hyaluronate and its derivatives for customized biomedical applications. Biomaterials.

[36] Williams DL, Wirostko BM, Gum G, Mann BK (2017). Topical cross-linked HA-based hydrogel accelerates closure of corneal epithelial defects and repair of stromal ulceration in companion animals. Invest. Ophthalmol. Vis. Sci..

[37] Fallacara A (2017). Novel artificial tears containing cross-linked hyaluronic acid: An in vitro re-epithelialization study. Molecules.

[38] Wong RS, Ashton M, Dodou K (2015). Effect of crosslinking agent concentration on the properties of unmedicated hydrogels. Pharmaceutics.

[39] Maiz-Fernandez S (2019). Synthesis and characterization of covalently crosslinked pH-responsive hyaluronic acid nanogels: Effect of synthesis parameters. Polymers (Basel).

[40] Lan SM, Jou IM, Wu PT, Wu CY, Chen SC (2015). Investigation into the safety of perineural application of 1,4-butanediol diglycidyl ether-crosslinked hyaluronan in a rat model. J. Biomed. Mater. Res. B Appl. Biomater..

[41] Frith JE (2013). An injectable hydrogel incorporating mesenchymal precursor cells and pentosan polysulphate for intervertebral disc regeneration. Biomaterials.

[42] Cowman MK, Lee HG, Schwertfeger KL, McCarthy JB, Turley EA (2015). The content and size of hyaluronan in biological fluids and tissues. Front. Immunol..

[43] Cyphert JM, Trempus CS, Garantziotis S (2015). Size matters: Molecular weight specificity of hyaluronan effects in cell biology. Int. J. Cell Biol..

[44] Levett PA, Hutmacher DW, Malda J, Klein TJ (2014). Hyaluronic acid enhances the mechanical properties of tissue-engineered cartilage constructs. PLoS One.

[45] Takeuchi K (2009). Solid hyaluronic acid film and the prevention of postoperative fibrous scar formation in experimental animal eyes. Arch. Ophthalmol..

[46] Takeuchi K (2011). Effects of solid hyaluronic acid film on postoperative fibrous scar formation after strabismus surgery in animals. J. Pediatr. Ophthalmol. Strabismus.

[47] Frangouli O, Adams GG (2013). The use of amniotic membrane for the management of fibrosis in complex strabismus surgery. Strabismus.

[48] Karamichos D, Guo XQ, Hutcheon AE, Zieske JD (2010). Human corneal fibrosis: An in vitro model. Invest. Ophthalmol. Vis. Sci..

[49] Gurumurthy S, Iyer G, Srinivasan B, Agarwal S, Angayarkanni N (2018). Ocular surface cytokine profile in chronic Stevens–Johnson syndrome and its response to mucous membrane grafting for lid margin keratinisation. Br. J. Ophthalmol..

[50] Schlunck G, Meyer-ter-Vehn T, Klink T, Grehn F (2016). Conjunctival fibrosis following filtering glaucoma surgery. Exp. Eye. Res..

[51] Li Y (2016). Effects of different sutures on fibrosis and wound healing in a rabbit model of corneal wounds. Exp. Ther. Med..

[52] Stramer BM, Zieske JD, Jung JC, Austin JS, Fini ME (2003). Molecular mechanisms controlling the fibrotic repair phenotype in cornea: Implications for surgical outcomes. Invest. Ophthalmol. Vis. Sci..

[53] Wirostko B, Mann BK, Williams DL, Prestwich GD (2014). Ophthalmic uses of a thiol-modified hyaluronan-based hydrogel. Adv. Wound Care (New Rochelle).

[54] Ronkart SN (2006). Determination of total water content in inulin using the volumetric Karl Fischer titration. Talanta.

[55] Gupta N, Kalaivani M, Tandon R (2011). Comparison of prognostic value of Roper Hall and Dua classification systems in acute ocular burns. Br. J. Ophthalmol..

[56] Yoeruek E (2008). Safety, penetration and efficacy of topically applied bevacizumab: Evaluation of eyedrops in corneal neovascularization after chemical burn. Acta Ophthalmol..

[57] Small BC, Murdock CA, Bilodeau-Bourgeois AL, Peterson BC, Waldbieser GC (2008). Stability of reference genes for real-time PCR analyses in channel catfish (*Ictalurus punctatus*) tissues under varying physiological conditions. Comp. Biochem. Physiol. B Biochem. Mol. Biol..

[58] Livak KJ, Schmittgen TD (2001). Analysis of relative gene expression data using real-time quantitative PCR and the 2(-Delta Delta C(T)) method. Methods.

