# Supplementary material for: Development of a novel hyaluronic acid membrane for the treatment of ocular surface diseases
Source: Sci Rep. 2021 Jan 27;11:2351. doi: 10.1038/s41598-021-81983-1 (PMC7840674; doi:10.1038/s41598-021-81983-1)

Development of a Novel Hyaluronic Acid Membrane for the Treatment of Ocular Surface Diseases

Dong Ju Kim, MD^1^, Mi-Young Jung^1^, Ha-Jin Pak^1^, Joo-Hee Park, PhD^2^, Martha Kim MD, PhD^1^, Roy S. Chuck MD, PhD ^3^, Choul Yong Park MD, PhD^1^

1. Department of Ophthalmology, Dongguk University, Ilsan Hospital, Goyang, South Korea

2. Department of Biochemistry, Dongguk University, College of Medicine, Gyeongju, South Korea

3. Department of Ophthalmology and Visual Sciences, Montefiore Medical Center, Albert Einstein College of Medicine, Bronx, NY, USA

**Supplementary Information**

**Full length gel of electrophoresis**

**Figure 6**


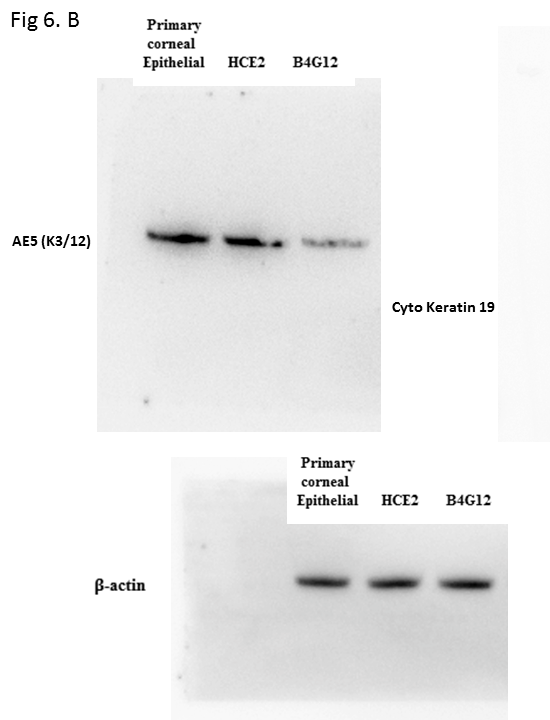


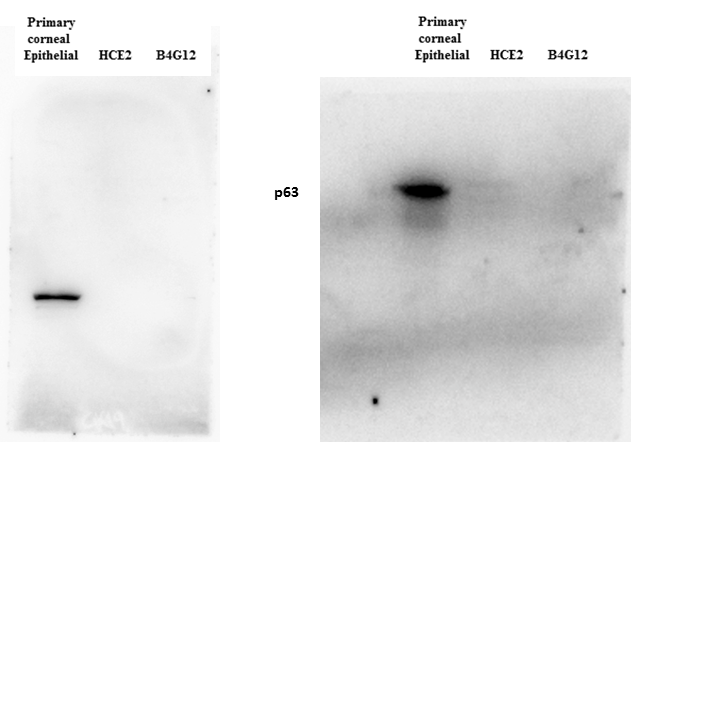


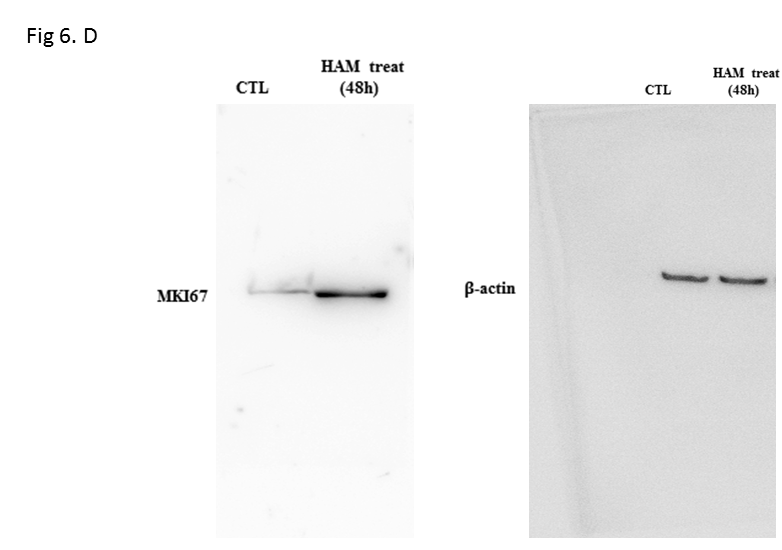

Supplement: Supplementary file 1 — Supplementary Information [file 41598_2021_81983_MOESM1_ESM.docx]
